# Outcomes of ventriculoperitoneal shunt insertion in the management of idiopathic intracranial hypertension in children

**DOI:** 10.1007/s00381-017-3423-0

**Published:** 2017-05-23

**Authors:** J. Heyman, Ronak Ved, A. Amato-Watkins, I. Bhatti, J. Te Water Naude, F. Gibbon, P. Leach

**Affiliations:** 1 Department of Paediatric Neurosurgery, University Hospital of Wales, Cardiff, UK; 2 Department of Neurosurgery, University Hospital of Wales, B4 Office, Cardiff, CF14 4XW UK; 3 Department of Paediatric Neurology, University Hospital of Wales, Cardiff, UK

**Keywords:** Paediatric, IIH, VP, Shunt

## Abstract

**Purpose:**

The ventriculoperitoneal (VP) shunt has become the procedure of choice for treatment of idiopathic intracranial hypertension (IIH). We aimed to assess the efficacy of frameless stereotactic placement of VP shunts for the management of medically resistant IIH in children and to assess the role of gender and obesity in the aetiology of the condition.

**Methods:**

This is a retrospective analysis of the case notes of 10 patients treated surgically at the University Hospital of Wales in Cardiff, from May 2006 to September 2012.

**Results:**

VP shunts were successful in relieving headache, papilloedema and stabilising vision. No sex predilection was identified, and increased BMI was a feature throughout the population, regardless of age.

**Conclusions:**

Neuronavigated VP shunt insertion is an effective mode of treatment for medically resistant IIH in children. The aetiological picture in children does not seem to be dominated by obesity, as in adults. Literature on childhood IIH is sparse, and larger scale, comparative studies would be of benefit to treating clinicians.

## Introduction

Ball et al. define idiopathic intracranial hypertension (IIH) as ‘the clinical syndrome of raised intracranial pressure in the absence of space-occupying lesions, vascular lesions or enlargement of the cerebral ventricles, for which no causative factor can be identified’ [1, 2]. Furthermore, patients who develop raised intracranial pressure (ICP) secondary to medications (e.g. doxycycline) or venous sinus stenoses are also classified as IIH patients [3, 4].

### Epidemiology and aetiology

Studies in North America have estimated that the incidence of IIH is 0.9–1.0 per 100,000 in the general population [2]. It is associated with female to male ratios ranging from 4:1 to 15:1 [4]. It is predominantly a condition of obese women of childbearing age, with an average age at diagnosis of 28.6 years [5, 6]. However, IIH also occurs in males, people of normal weight and children [7]. The incidence of IIH in children is unknown due to the approach of previous studies; paediatric patients are typically mixed in within adult study populations, and there is a lack of exclusion of patients for which secondary aetiology was found [8–10]. Therefore, there is little evidence to support the hypothesis that those risk factors associated with IIH in adults hold true for paediatric patients. Cinciripini et al. found that IIH in pre-pubertal children is rare, that there was no sex predilection and that obesity was uncommon [5].

### Diagnosis and treatment

IIH is a syndrome characterised by the symptoms and signs of raised ICP, e.g. headache and blurred vision with papilloedema. The most frequent presenting symptom in children is headache [11, 12]. Visual disturbance is also common, with many patients developing transient visual obscurations, blurring and/or progressive loss of peripheral vision. These progressive ophthalmological problems are the result of papilloedema and can lead to blindness if left untreated. Less commonly, patients can present with diplopia or pulsatile tinnitus [13]. Criteria for the diagnosis of IIH were originally proposed by Dandy [14] in 1937, revised by Friedman and Jacobson [2] in 2002 (Table 1).

**Table 1:** Modified Dandy criteria for the diagnosis of IIH in both adults and children as revised by Friedman and Jacobson [2, 14]

Modified Dandy criteria for the diagnosis of idiopathic intracranial hypertension
1. If symptoms present, they may only reflect those of generalised intracranial hypertension or papilloedema
2. If signs present, they may only reflect those of generalised intracranial hypertension or papilloedema
3. Documented elevated intracranial pressure measured in the lateral decubitus position
4. Normal CSF composition
5. No evidence of hydrocephalus, mass, structural or vascular lesion on MRI or contrast-enhanced CT for typical patients, and MRI and MR venography for all others
6. No other cause of intracranial hypertension identified

Weight loss, discontinuation of precipitating drugs and pharmacological management with acetazolamide (carbonic anhydrase inhibitor) and other diuretics are the first line treatments for IIH. Lumbar puncture aids diagnosis in addition to therapeutically reducing ICP and providing symptom control. Surgical intervention is typically reserved for those who respond poorly to or fail to tolerate medical treatment or in those whom vision is acutely deteriorating. The surgical interventions employed to treat medically refractory IIH include optic nerve fenestration, ventriculoperitoneal (VP) or lumboperitoneal (LP) shunt insertion and intracranial venous sinus stent deployment. Based on currently available evidence, there is insufficient data to confidently recommend or reject any of the surgical treatments modalities for IIH [15].

Nevertheless, shunting procedures are most commonly used, and provide rapid and effective reduction in intracranial pressure. Lumboperitoneal shunts were previously favoured as ventricles in IIH patients are often very small, making shunt insertion difficult. However, LP shunts are associated with a high complication rate [13] and with the development of frameless stereotactic navigation (FSN), which has been shown to increase the chances of optimal positioning of the proximal catheter [9, 16, 17]; VP shunt insertion utilising FSN has become the procedure of choice.

## Materials and methods

Patients under the age of 18 years, who fulfilled the modified Dandy criteria [14] for the diagnosis of IIH, that had undergone a neuronavigated VP shunt insertion at our institution from May 2006 (when Stealth technology was acquired in our department) to September 2012 (*n* = 10) were retrospectively analysed. Intraoperative image guidance was achieved using a wand-based navigation system (Stealth Station, Medtronic Inc. ©). After the registration process, the target site for the catheter was determined, with the Foramen of Munro used for all cases, in line previous studies [18, 19]. The operative details of FSN-guided VP shunt insertion have been described [9, 18, 19, 20]. A standard procedure for all 10 patients, whereby a nine-gauge catheter with the stereotactic wand inserted was advanced towards the target using image guidance, was followed.

Data was collected regarding demographics, clinical presentation, details of treatment, alleviation of symptoms, ophthalmological examinations and length of follow-up.

When logging the visual acuity data, each eye of the 10 patients was analysed individually. LogMAR scores were used to quantify visual acuity. When testing the acuity of children, their score is dependent on co-operation during the examination. To allow for this subjectivity, patients must record a difference of ≥0.2 on the LogMAR scale to be considered as having a significant change in their visual acuity.

The pre-operative visual acuity data for one patient (patient 10) could not be retrieved, and therefore, the pre to post-operative difference could not be analysed. However, in order to not discount this patient fully from our study, and so that the procedure’s effect on visual acuity could be assessed, three of the most recent visual acuity tests post-operatively were analysed. This allowed us to gauge whether this patient had achieved a degree of visual benefit from the procedure.

BMI data was calculated for all 10 patients and was used to identify a *z*-score. This enabled analysis based on the standard deviation from the age-specific median weight for each patient. Using the World Health Organisation (WHO) BMI-for-age criteria [21], it was identified whether the patient lay in the category of obese (>98th percentile), overweight (91st-97th percentile), normal weight (2nd-90th percentile), low weight (1st-2nd percentile), or very low weight (<1st percentile).

## Results

Six patients were male and four were female. Mean age at diagnosis was 11.5 years (range 6–17). Follow-up time ranged from 12 to 59 months, with a mean of 30.6 months (Table 2).

**Table 2:** Summary of the patient demographics and clinical characteristic data

Patient number	Age at diagnosis	Age at op in years	Sex	Headache before	Headache after	Papilloedema before	Papilloedema after	Visual acuity after	Body mass index	Follow-up in months
1	14	14	F	Yes	Yes	No	No	Stable	27	59
2	11	11	M	Yes	No	Yes	No	Stable	21	38
3	7	9	M	Yes	No	Yes	No	Stable	21	36
4	17	17	F	Yes	No	Yes	No	Stable	25	33
5	9	11	M	Yes	No	Yes	No	Stable	18	34
6	12	12	M	Yes	Yes	Yes	No	Stable	30	29
7	10	11	M	Yes	No	Yes	Yes	Decreased	25	26
8	6	6	F	Yes	No	Yes	No	Improved	14	23
9	12	12	M	Yes	No	Yes	No	Stable	29	16
10	17	17	F	Yes	No	Yes	Yes	Decreased in R eye, stable in L	26	12

### Headache

All 10 patients reported headache pre-operatively. Eight patients reported no headache at their most recent follow-up.

### Papilloedema

Nine patients (90%) had evidence of papilloedema at presentation. Of these, eight (80%) had no evidence of papilloedema post-operatively.

### Visual acuity

The LogMAR scores for the patients whose pre- and post-operative scores were available (*n* = 9) are displayed in Table 3. Included in the table is an indication of whether the visual acuity has improved (∧), deteriorated (∨) or remained stable (–). When reading LogMAR data, it must be remembered that a positive change in the score represents deterioration in visual acuity whereas a negative change represents an improvement.

**Table 3:** Data on pre- and post-operative visual acuities

Patient number	Pre-operative		Post-operative	
	Right eye	Left eye	Right eye	Left eye
1	−0.18	−0.18	+0.00 –	−0.18 –
2	+0.00	−0.04	−0.10 –	+0.08 –
3	+0.00	+0.00	−0.08 –	−0.08 –
4	+0.00	+0.00	+0.18 –	+0.18 –
5	+0.12	+0.12	+0.00 –	+0.00 –
6	+0.14	+0.14	+0.02 –	+0.1 –
7	+0.30	+0.18	+1.60 ∨	+1.16 ∨
8	+0.30	+0.30	+0.00 ∧	+0.00 ∧
9	+0.30	+0.48	+0.48 –	+0.65 –

**Fig. 1:**
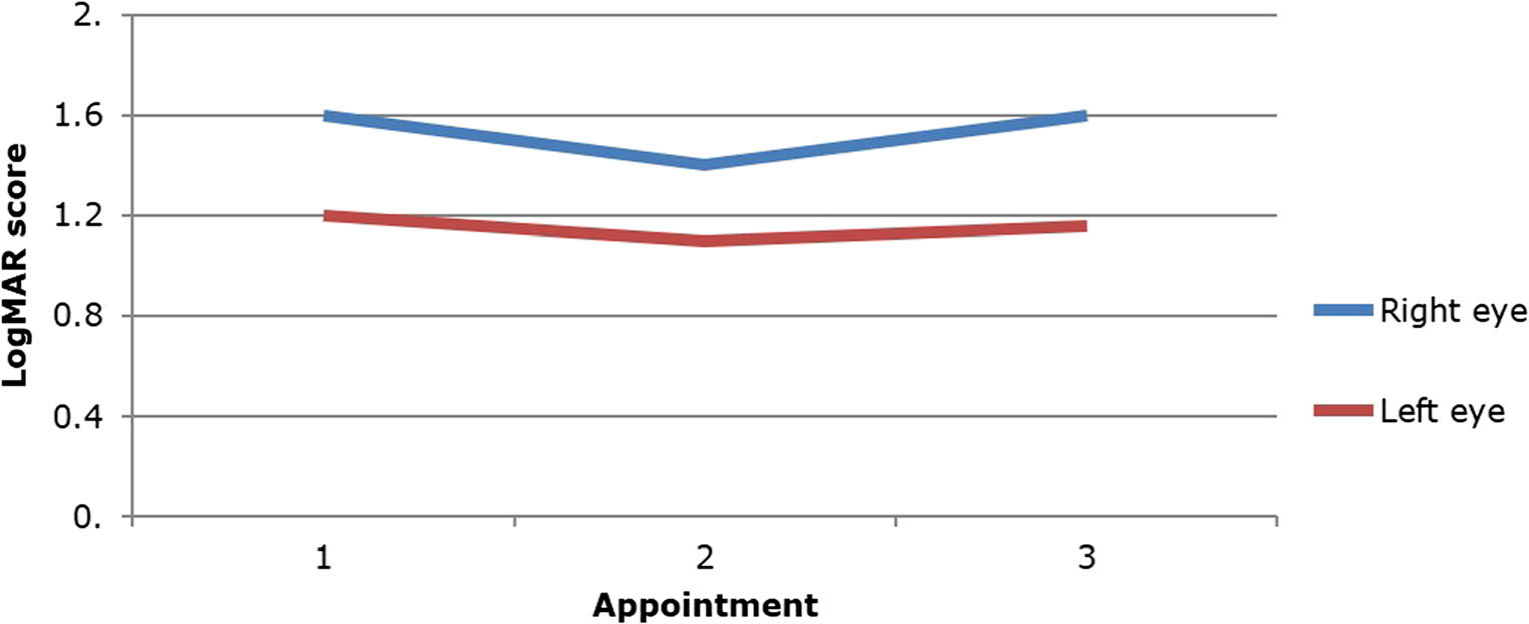
Visual acuity for patient 7 post-operatively showing stable vision once a functioning shunt system was achieved (appointment 3). *Blue line*, right eye; *red line*, left eye

The visual deterioration seen in patient 7 was secondary to multiple shunt malfunctions over a 4-month period. However, once an effectively working shunt was in place, this patient’s vision stabilised (Fig. 1).

**Fig. 2:**
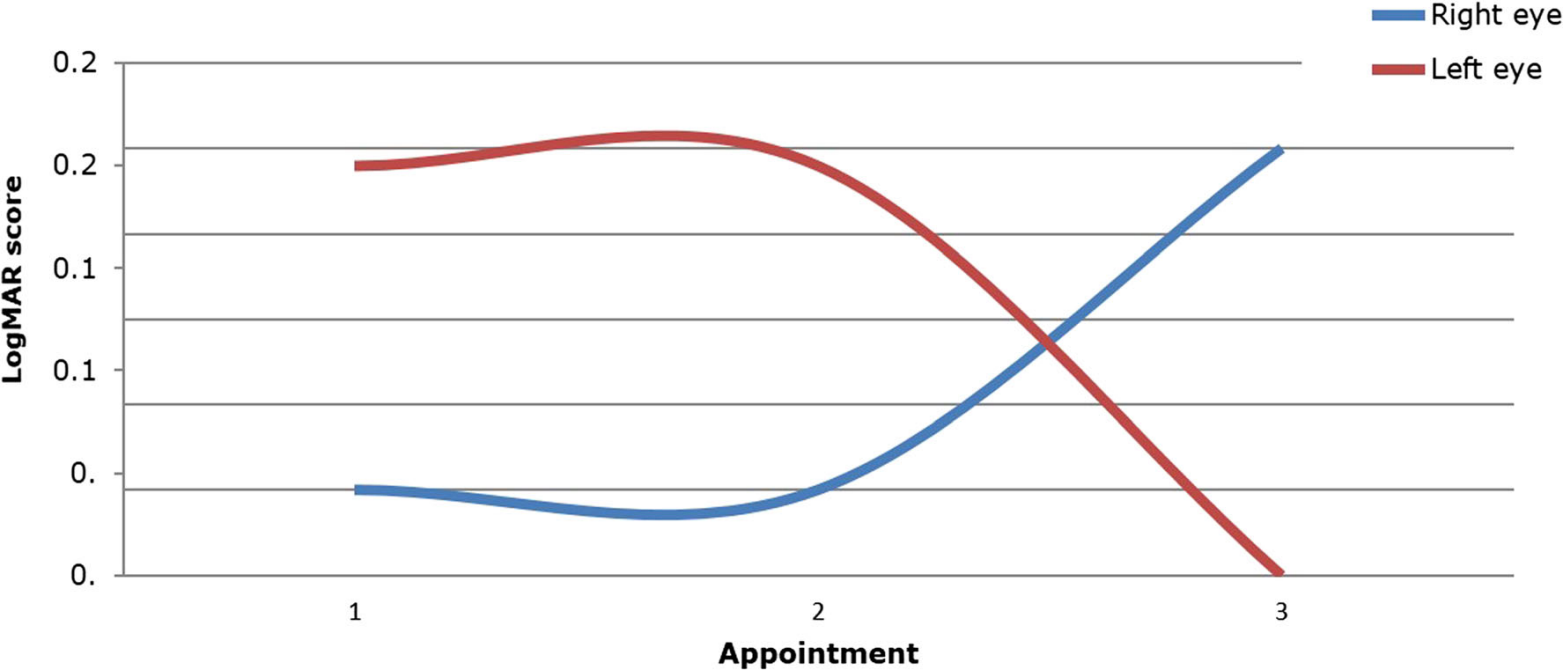
Line graph displaying post-operative LogMAR data for patient 10 for the latest three eye examinations post-shunt insertion. Visual acuity in the left eye stabilised, whereas that of the right eye deteriorated, despite clinical confirmation of a functioning VP shunt. *Blue line*, right eye; *red line*, left eye

The pre-operative LogMar score for patient 10 could not be retrieved. However, the most recent three post-operative visual acuity scores were used to assess the patient’s visual acuity. These showed that the left eye had stabilised, but the right had deteriorated (Fig. 2). This patient subsequently underwent ICP monitoring which revealed normal ICP and therefore an adequately functioning VP shunt.

### Lumbar puncture opening pressure

Opening pressure readings from the patients’ pre-operative lumbar punctures ranged from 35 to 75 cm H_2_O. The mean value was 46.6 cm H_2_O. Indication of whether the visual acuity has improved (∧), deteriorated (∨) or remained stable (–) is shown (Table 4).

**Table 4:** Pre-operative lumbar puncture opening pressures, alongside symptom improvement after lumbar puncture data

Opening pressure (cm H_2_O)	Headache relief	Papilloedema relief	Visual acuity improvement/deterioration—R eye	Visual acuity improvement/deterioration—L eye
35.0	Y	Y	–	–
37.0	N	N/A	–	–
39.5	Y	Y	–	–
40.0	Y	Y	–	–
40.0	N	Y	–	–
40.0	Y	Y	–	–
40.0	Y	N	N/A	N/A
49.0	Y	Y	–	–
70.0	Y	Y	∧	∧
75.0	Y	N	∨	∨

*N/A* not available

### Body mass index

**Fig. 3:**
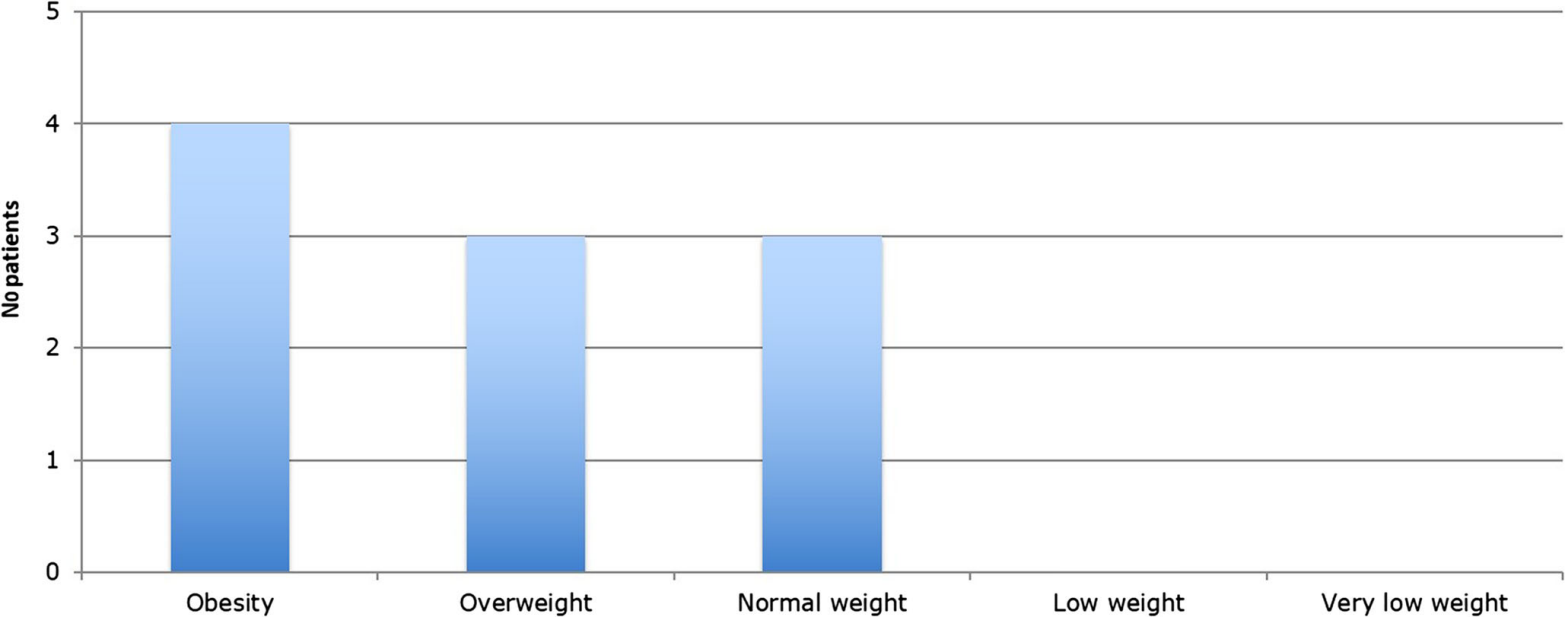
Data on the number of patients in each weight classification (*n* = 10)

Data for the number of patients that fell into each WHO BMI-for-age weight category is shown below (Fig. 3). Forty percent of patients were classed as obese (*n* = 4); 30% were classed as overweight (*n* = 3); ergo, 70% of the study population were classed as above a normal weight category. Patients were also split into 0–11 years (*n* = 5) and 12–17 years (*n* = 5) of age at time of diagnosis. In both age categories, 40% of the patients were obese (*n* = 2 in each age group). The younger age group contained 40% within the normal weight category (*n* = 2), whereas the older group contained only one child with a normal weight (20%; Fig. 2). No patients in the entire cohort had low or very low weights.

## Discussion

This study of ten paediatric patients with IIH has assessed the role of gender and obesity in the aetiology of the condition, and provided outcome data supporting the efficacy of frameless stereotactic placement of VP shunts for the management of medically resistant IIH in children.

### Demographics

The adult female sex predilection of IIH was not found in this paediatric study, as 60% (*n* = 6) of patients were male. Other studies have also failed to identify a clear female sex predilection in younger patients with paediatric IIH [1, 3, 4]. However, the literature has identified that older children with IIH may display associations with obesity and the female sex, mirroring the established associations of adult IIH [3]. It has tentatively been postulated that this difference in the demographics of paediatric IIH patients may be related to whether patients are pre- or peri-pubertal. This notion is supported by a number of case reports linking the onset and/or resolution of paediatric IIH with imbalances in a variety of hormones, including thyroid hormone, corticosteroids, oestrogens and growth hormone [9]. Collating further evidence from larger case series incorporating analysis of patients’ endogenous hormonal states could uncover whether there may be etiologically distinct mechanisms underlying IIH, depending on whether onset is prior to, during or post-pubertal [9, 10, 22].

### Headache

Rangwala et al. state that headache is the single most common complaint among children with idiopathic intracranial hypertension and is present in 62–91% of cases [12]. This is reflected in our study, with all patients reporting pre-shunt headache. Eighty percent of patients (*n* = 8/10) described no headache at their latest follow-up appointment.

### Papilloedema

Nine (90%) of our patients had papilloedema pre-operatively, and eight of these patients had no papilloedema at their most recent follow-up. The remaining patient showed improvement but not complete resolution of papilloedema. It is, however, recognised that some patients have continuing papilloedema with a functioning shunt. Tulipan et al., for example, found that 29% of their patients still showed evidence of papilloedema post-shunt [20]. This may reflect the chronicity of the condition in a sub-group of patients causing irreversible damage to the optic nerve or be a consequence of continued elevation of ICP, requiring further treatment. Meticulous fundoscopy is essential during post-operative follow-up to identify patients who may require further surgery as early as possible.

### Visual acuity

The decline in vision poses a significant problem in IIH, with long-term consequences for the patient. Indeed, progressive loss of vision is the principal criteria for surgical intervention. Of the nine patients for whom we had paired pre- and post-operative visual acuity data, visual acuity stabilised in both eyes after shunt insertion in 89% of patients (*n* = 16/18 eyes).

One patient had a series of shunt malfunctions after insertion that caused visual deterioration not noticed by the child or parents. This was identified at his first clinical review post-operatively at 3 months. His vision stabilised once an adequately working shunt was established (Fig. 1). The one patient with only post-operative eye assessments showed improvement in the left eye but deterioration in the right eye, even though her intracranial pressure was normal (Fig. 2). This may be a consequence of severe, irreversible damage to the optic nerve in this patient’s right eye pre-operatively.

### Body mass index

In adults, there is a well-established association between obesity and IIH [11]. The link between obesity and children with IIH is less clear, and there are discrepancies in the literature regarding this. When dealing with paediatric study populations, Babikian et al. found that 33% were obese [1], whereas 70% were obese in the study performed by Balcer et al. [3]. Though most studies agree that the role of obesity may not be as pronounced as it is in adults [3], there is no consistent quantification of the relationship between obesity and IIH in children.

Balcer et al. separate their paediatric subject population into tertiles by age in order to examine the relationship between IIH and obesity at varying ages. They found that older children with IIH were more likely to be obese than younger children—between the ages of 3–11 years, 41% of patients were obese, whereas this number increased to 91% in the 15–17 years age group [3]. Balcer et al. state that in order to discover the true aetiology, we must ‘examine the potential roles of metabolic, hormonal, or other physiologic factors’ [3, 20].

In both age categories in our study (0–11 *n* = 5; 12–17, *n* = 5), 40% of patients were obese. The younger age group contained 40% within the ‘normal’ weight category, whereas the older group contained only 20%. Only 40% of all patients in the entire cohort were classed as ‘obese’. This study therefore echoes the suggestion postulated by previous works that the role of obesity may not play as significant a role in the paediatric population as it does in adult IIH. However, a further 30% of the entire cohort (*n* = 3) was classed as ‘overweight’, resulting in a majority of our population (70%) being classed as over the normal weight category. This may serve to strengthen the hypothesis put forward by Daniels et al. that it is not specifically obesity, but an absolute high BMI in general which could be an important associated factor with paediatric IIH [6].

### LP opening pressure

The data on LP opening pressure failed to suggest a link between pre-operative pressure and the relief of either symptom or papilloedema, although we appreciate that the numbers are small.

### Use of frameless stereotactic neuronavigation

Frameless stereotactic neuronavigation has been shown to improve the chances of optimal placement of ventricular catheters when compared to conventional shunt surgery, with successful ventricular cannulation typically reaching 100% when the technology is utilised [9, 16, 18, 21]. Whilst its use may slightly increase operative time [23], there appears to be no difference in infection rates, and the revision rates are significantly lower than those for LP shunts [9, 23]. In one series of paediatric patients with slit ventricles, the use of FSN aided successful ventricular cannulation in 100% of the patient cohort, with zero shunts requiring revisions at 8 months post-operatively [8].

VP shunt revision rates after conventional shunt placement procedures ranges between 30 and 50% at 24 months [18, 21, 23]. In a case series analysing FSN, revision rates were similar or lower for patients with IIH (e.g. 30% at 49 months [9]) when compared to a series of conventionally inserted VP shunts [9, 18, 21]. The important caveat here is that the historical revision rate data is largely based upon blind cannulation of hydrocephalic ventricles, as opposed to the more challenging slit-like ventricles typical of IIH. Furthermore, the proportion of shunt revisions due to proximal catheter obstruction appears to be lower when FSN is used to aid insertion [18, 21]; distal obstruction/migration and under/overdrainage appear to account for the majority of shunt revision procedures when FSN is used at the first operation.

In reviews of clinical effectiveness after FSN-guided VP shunt insertion for IIH, significant improvements in headache, stabilising vision and/or reduction in papilloedema, have been reported [8, 16, 18, 21, 24]. These findings have been corroborated by data from this study, which has also confirmed that these improvements in headache and visual deterioration can be sustained over longer follow-up periods (12–59 months) than in previous series (8–9 months).

### Study strengths and limitations

This is a retrospective case series of a rare condition. This is illustrated clearly by the fact that in South Wales, there have been only 10 paediatric cases requiring surgical treatment (VP shunt insertion) during the 76-month study period. On review of the literature, comparable studies involve very similar numbers, which are almost certainly a function of the relative rarity of the condition in the paediatric population. For example, the studies performed by Cinciripini et al. and Bynke et al. had only 10 and 17 patients, respectively [2, 20, 25].

An established criterion for surgical intervention in paediatric IIH is threat to, or deterioration of, vision in the presence of failure of medical therapy [13, 15]. Our data identifies that frameless navigated VP shunt insertion is an effective and safe surgical option. The high rates of LP shunt failure and peri-operative complications [24] therefore make stereotactic VP shunting an attractive alternative method for CSF diversion in IIH. Optic nerve fenestration remains another surgical option in the management of severe visual loss in IIH. However, it does not provide relief from headaches, and neither does it guarantee preservation of vision [10, 12]. Most studies thus far have focused on paediatric IIH case series’ outcomes after optic nerve fenestration and LP shunting [9, 16, 17]. Our study highlights the relative safety and effectiveness of image guided VP shunting in paediatric patients, and thus a comparison of outcomes after stereotactic VP shunts and optic nerve fenestration in paediatric IIH is required.

## Conclusion

Frameless navigation for VP shunt placement is an effective method of managing medication-resistant IIH in children for both symptomatic relief and stabilisation of visual acuity. The aetiological picture in the pubertal and post-pubertal paediatric population is likely to be similar to that of adults with regard to an association with an above-normal BMI. But this notion falls short of the obesity-dominated aetiology that is well documented in the adult population, particularly in younger children. The female sex predilection seen in adult patients does not seem to be present in young children with IIH. Furthermore, children often do not notice deterioration in their vision, and therefore, the authors suggest early clinical and ophthalmological reviews after shunt insertion to identify possible shunt malfunction.
